# Cardiac Schwannoma: A Clinical Case Report and Diagnostic Perspectives

**DOI:** 10.70352/scrj.cr.25-0300

**Published:** 2025-09-23

**Authors:** Peipei Zhang

**Affiliations:** Department of Ultrasound, The First Affiliated Hospital of Shandong First Medical University & Shandong Provincial Qianfoshan Hospital, China

**Keywords:** cardiac schwannoma, case report, diagnostic perspectives

## Abstract

**INTRODUCTION:**

Primary cardiac schwannoma (PCS) is an exceptionally rare neurogenic tumor, with only 53 documented cases worldwide.

**CASE PRESENTATION:**

We report the case of a 62-year-old woman with a 3-year history of paroxysmal palpitations, who subsequently developed progressive symptoms, such as chest tightness, dyspnea, and dizziness. Diagnostic evaluation revealed supraventricular tachycardia along with a 3.4 × 3.5 cm mass located at the coronary sinus ostium. Multimodal imaging—including echocardiography, CT, and MRI—revealed features characteristic of schwannoma, such as T1 hypointensity, T2 hyperintensity, and peripheral enhancement with internal septations. The patient underwent complete surgical resection of an encapsulated interatrial septal mass measuring 5.0 × 4.0 cm via a right atrial approach, with preservation of cardiac architecture and maintenance of compensatory venous drainage through an accessory ostium. Histopathological analysis confirmed a benign schwannoma exhibiting secondary degenerative changes, supported by positive SOX-10 and S-100 immunostaining and a low Ki-67 proliferation index. At the 3-year follow-up, the patient remained free of recurrence and exhibited no postoperative complications.

**CONCLUSIONS:**

This case underscores the diagnostic challenges associated with PCS, particularly in the context of arrhythmias, and emphasizes the importance of multimodal imaging in the preoperative evaluation. The successful surgical resection demonstrates the importance of meticulous surgical planning and technique in managing these rare tumors. This report adds to the limited body of literature on PCS and reinforces the need for a multidisciplinary approach in the diagnosis and management of these complex cases.

## Abbreviation

PCS
: primary cardiac schwannoma

## INTRODUCTION

PCS is an exceedingly rare Schwann cell-derived neurogenic tumor, with only 53 cases reported worldwide, of which 17 are within the cardiac chambers (atria or ventricles).^1)^ These tumors most commonly involve the right atrium, right ventricle, and left atrial wall.^1,2)^ Clinical manifestations can range from incidental findings to symptoms such as chest pain, syncope, and heart failure; rare cases have presented with intrapericardial hemorrhage mimicking acute coronary syndrome.^3)^ Imaging modalities such as echocardiography, CT, and MRI are essential for distinguishing schwannomas from other cardiac neoplasms. Histopathological examination typically shows spindle-shaped Schwann cell-like elements, with S100 positivity and a low Ki-67 index, consistent with benign features. However, rare malignant transformations have been reported.^4,5)^ Surgical resection remains the primary treatment and is successful in most cases, with long-term surveillance recommended due to recurrence and transformation risks.

## CASE PRESENTATION

**Fig. 1:**
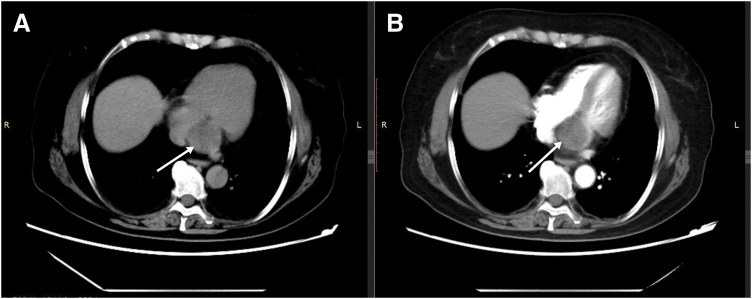
Contrast-enhanced CT scans. (**A**) Non-contrast image showing a round, heterogeneous hypodense lesion approximately 4.2 cm in diameter within the interatrial septum, with an arrow pointing to the mass. (**B**) Contrast-enhanced image demonstrating mild enhancement of the mass with partial protrusion into the left atrium; the arrow indicates the tumor.

**Fig. 2:**
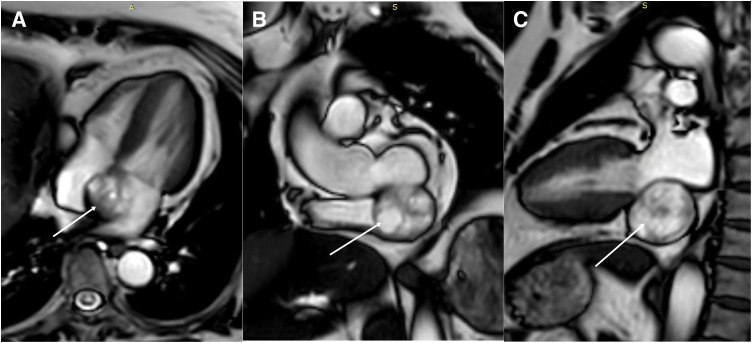
Contrast-enhanced MRI. (**A**) A round lesion with heterogeneous hyperintensity on T1- and T2-weighted sequences at the coronary sinus ostium in the left atrioventricular groove region; the arrow highlights the lesion. (**B**) Post-contrast images exhibit peripheral enhancement with internal septations; the central area remains non-enhancing, arrow indicating the tumor. (**C**) Well-defined margins of the lesion are clearly delineated; the arrow points to the mass.

A 62-year-old woman with a 3-year history of paroxysmal palpitations presented to our institution with worsening symptoms over 5 days, characterized by increased episode frequency, chest tightness, dyspnea, dizziness, and eyelid heaviness, notably without syncope, visual disturbances, chest pain, or loss of consciousness. Electrocardiography revealed sustained tachycardia consistent with supraventricular tachycardia (SVT). Transthoracic echocardiography identified a well-demarcated, isoechoic, round mass measuring 3.3 × 3.1 cm, situated adjacent to the coronary sinus within the left atrioventricular groove, causing complete obliteration of the sinus architecture. No significant impact of the tumor on the tricuspid or mitral valves was observed. Importantly, there was no evidence of hemodynamic compromise; cardiac chamber function was preserved. Contrast-enhanced thoracic CT demonstrated a 4.2 cm heterogeneous hypodense lesion (mean attenuation: 25 Hounsfield units [HU]) within the interatrial septum, with mild contrast enhancement. A slight leftward extension into the left atrial cavity was observed (**Fig. 1**). Cardiac MRI revealed a well-circumscribed, 3.4 × 3.5 cm mass at the coronary sinus ostium (left atrioventricular groove), characterized by T1 hypointensity, T2 hyperintensity, and heterogeneous signals on fat-saturated T2 sequences. Post-contrast imaging showed peripheral enhancement with internal septal enhancement and a central non-enhanced zone (**Fig. 2**).

**Fig. 3:**
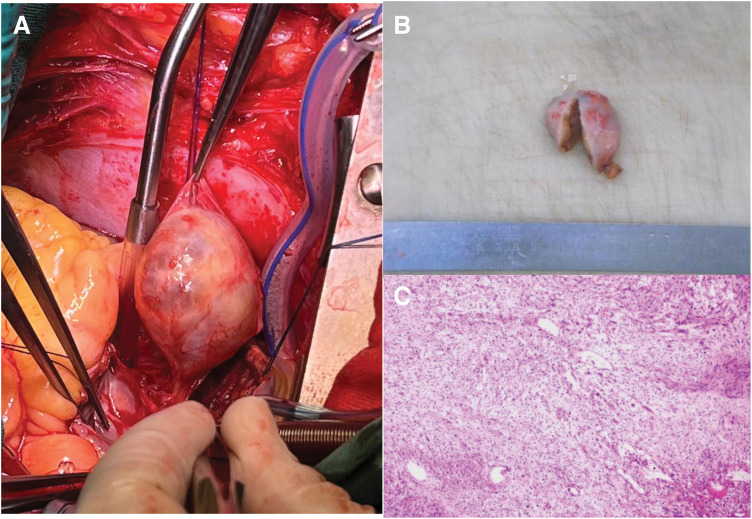
(**A**) Intraoperative view showing a round, encapsulated mass with a slender pedicle. (**B**) Gross examination: a single tumor measuring 4.6 × 4.1 × 2.4 cm with a smooth, encapsulated surface; the cut surface reveals a cystic-solid architecture with cystic spaces (0.4–1.1 cm) containing clear fluid; solid areas are gray-white, gray-yellow, and gray-red with a firm consistency. (**C**) Hematoxylin and eosin staining showing positivity for SOX-10 and S-100; CD34 highlights vascular components; Desmin is negative; and Ki-67 proliferative index is approximately 1%.

The patient underwent complete resection of a 5.0 × 4.0 cm encapsulated interatrial septal mass originating from the muscular layer anterior to the fossa ovalis, attached by a 3-mm pedicle (**Fig. 3A**). The tumor exerted significant compressive effects on adjacent structures, including inferior displacement of the coronary sinus with formation of a 1-cm accessory ostium, anterior impingement on the tricuspid annulus and conduction system, and posterior abutment of the Eustachian ridge. A right atrial approach was used to access the tumor, with careful dissection along the atrial septum to expose the mass. The atrial septum on the side of the tumor was incised, approximately 5 cm in length, revealing the tumor in the subendocardial layer of the left atrium, without penetration into the left atrial endocardium. It was determined that the tumor originated from the septal muscle layer. The most prominent part of the tumor on the right atrial side was resected with a transverse incision of about 4 cm. The tumor was separated from the subendocardial muscular layer using a scalpel and cautery, with 5-mm muscular margins around the pedicle to ensure complete resection. The tumor capsule was intact, oval-shaped, and milky white, with a stalk about 3 mm in diameter originating from the anterior myocardial layer of the oval fossa of the atrial septum. The surrounding 5 mm of muscular tissue was excised along with the stalk.

The resected tumor, measuring 4.5 × 3.5 cm, was whitish, rubbery, and intact, without penetration into the left atrium. Concurrent reconstruction of the coronary sinus and septum preserved normal cardiac architecture and maintained the accessory venous ostium for effective venous drainage. To achieve this, a continuous 5-0 Prolene sutures (Ethicon, Somerville, New Jersey, USA) was used to repair the septal defect, reshaping the atrial septum. The coronary sinus opening’s endocardium was meticulously re-sutured and fixed at the Koch triangle using fine suturing techniques to ensure the integrity of the venous pathway. After reshaping, the perfusion stopped, and good return flow was observed from the coronary sinus opening. The atrial septum and right atrial incision were sutured. A pacemaker lead was placed on the epicardial surface, ensuring proper pacing and monitoring during the postoperative recovery period.

Histopathological analysis revealed a solitary, encapsulated cardiac tumor with a smooth surface measuring 4.6 × 4.1 × 2.4 cm. Histological examination showed a cystic-solid pattern. Immunohistochemistry confirmed a benign schwannoma with secondary degenerative changes, evidenced by SOX-10 and S-100 positivity and a low Ki-67 proliferation index (**Fig. 3B**, **3C**). The postoperative electrocardiogram showed no significant abnormalities. The patient’s postoperative course was uneventful, with the length of hospitalization extending to 23 days. At 3 years postoperatively, the patient remained in sustained recovery, with no evidence of tumor recurrence or procedure-related complications.

## DISCUSSION

This case underscores the diagnostic challenges of PCS presenting with arrhythmias. Initial detection relied on echocardiography, which served as the screening tool; however, cross-sectional imaging—particularly CT and MRI—provided essential discriminative features. Specifically, encapsulation and septal enhancement observed on imaging strongly suggested a schwannoma, which was subsequently confirmed through histopathological examination. The tumor’s proximity to the coronary sinus and its distinctive imaging characteristics contributed to the diagnostic complexity, underscoring the need for detailed discussion. The complementary use of multiple imaging modalities, from echocardiography to CT and MRI, proved crucial in characterizing the tumor’s features and extent. The hallmark imaging characteristics—T1 hypointensity, T2 hyperintensity, and peripheral enhancement with internal septations—were strongly suggestive of a schwannoma, which was later confirmed histopathologically.^6)^ These findings are consistent with previous reports, emphasizing the vital role of multimodal imaging in diagnosing cardiac schwannomas.

The patient’s initial presentation with paroxysmal palpitations and subsequent supraventricular tachycardia highlights the importance of evaluating underlying structural cardiac abnormalities in cases of refractory arrhythmias.^7)^ The tumor’s proximity to the conduction system, especially its anterior compression of the tricuspid annulus, likely contributed to electrical instability. Although previous reports have described associations between cardiac schwannomas and arrhythmogenesis, the exact mechanisms remain poorly understood.

The surgical technique demonstrated that complete resection is feasible even in anatomically challenging regions. A right atrial approach enabled successful enucleation with adequate margins, preserving cardiac structure. Preservation of the accessory venous ostium for venous drainage exemplifies strategic planning that likely contributed to the patient’s favorable postoperative course. Although the primary coronary sinus ostium was already reopened during tumor resection, maintaining the accessory ostium may still confer benefits. First, it preserves an alternative drainage channel for posteroinferior myocardial veins, thereby safeguarding against localized venous hypertension should pericoronary fibrosis or edema develop. Second, avoiding suture closure near the accessory ostium reduces manipulation of the underlying atrioventricular conduction tissue, potentially lowering the risk of postoperative arrhythmias. The absence of tumor recurrence at 3 years post-surgery further supports the effectiveness of this approach.

## CONCLUSIONS

This case highlights the role of multimodal imaging in the diagnosis and preoperative evaluation of PCS. The successful surgical resection of the tumor, even in a challenging anatomical location, demonstrates the importance of careful surgical planning.
